# Research on the application and effect of flipped-classroom combined with TBL teaching model in WeChat-platform-based biochemical teaching under the trend of COVID-19

**DOI:** 10.1186/s12909-023-04623-4

**Published:** 2023-09-19

**Authors:** Haiyan Ji, Kangle Zhu, Zhiyu Shen, Huixia Zhu

**Affiliations:** 1 Nantong University, Nantong, Jiangsu Province 226001 China; 2 Nanjing Drum Tower Hospital Clinical College of Nanjing Medical University, Nanjing, Jiangsu Province 211166 China

**Keywords:** COVID-19, WeChat, Biochemistry, Blended teaching, Teaching effect

## Abstract

**Background:**

Biochemistry is a core subject in clinical medical education. The traditional classroom teaching model led by teachers is often limited to the knowledge transfer of teachers and the passive acceptance of students. It lacks interactive and efficient teaching methods and is not enough to meet the learning needs and educational goals of modern students. The combination of WeChat public platform, flipped classroom and TBL teaching model is closer to the needs of real life and workplace, helping students to cultivate comprehensive literacy and the ability to solve practical problems. At the same time, this teaching model has yet to be used in biochemistry courses.

**Objective:**

To explore the influence of the mixed teaching model of flipped classroom and combining TBL based on WeChat public platform upon undergraduates in biochemistry.

**Methods:**

Using the mixed research method of quasi-experimental research design and descriptive qualitative research, 68 students were selected into the traditional and the blended teaching groups. Among them, the blended teaching group adopts the blended teaching model of flipped classroom combined with TBL based on the WeChat platform to learn biochemical courses. In this study, an independent sample t-test was intended to analyze the differences in final scores, a chi-square test was served to analyze the differences in satisfaction questionnaires, and thematic analysis was used to analyze semi-structured interview data.

**Results:**

Compared with the traditional teaching model, the mixed teaching model significantly improved students' final exam scores (*P* < 0.05). The teaching satisfaction of the mixed teaching group was also higher than that of the traditional teaching group with statistical significance (*P* < 0.05). The results of the interviews with eight students were summarized into three topics: (1) Stimulating interest in learning; (2) Improving the ability of autonomous learning; (3) Recommendations for improvement.

**Conclusions:**

The combination of the WeChat platform and flipped classroom with TBL has a positive effect on improving medical students' autonomous learning ability and problem-solving ability. The research shows that the teaching mode of flipped classroom combined with TBL based on the WeChat platform is effective and feasible.

## Introduction

Biochemistry is a core discipline in clinical medicine education, as well as the most rapidly progressing and dynamic frontier in the life sciences. As a life science, biochemistry deeply explores the chemical processes and molecular mechanisms in living organisms and provides basic ideas for clinical medicine to deeply understand and explain the occurrence, development, and treatment of diseases. Therefore, biochemistry is a bridge between basic and clinical medicine and an essential course that must be mastered by clinically highly qualified personnel. And the cultivation of highly skilled clinicians requires not only students to master basic knowledge such as biochemistry but also that they can think independently, learn autonomously, and solve practical problems [1–3].

With the rapid development of biochemistry theoretical knowledge and practical technology, research in related fields is increasing with astonishing speed. Due to the wide range of knowledge covered by biochemistry, the concepts and mechanisms often take time to understand. However, teachers mainly teach traditional Lecture-based Learning (LBL), and students can only passively accept knowledge [4]. As a result, it is difficult for students to comprehend a lot of basic knowledge in a limited time. In addition, the traditional teaching model needs more teacher-student and student–student interaction in the classroom, which may not be conducive to cultivating students' autonomous learning ability and teamwork ability, significantly increasing the difficulty of training high-quality clinicians [5, 6]. Therefore, it is urgent to find and build a new teaching model to improve students' learning efficiency, independent learning ability and teamwork ability.

Flipped Classroom (FC) is a new teaching model that rearranges the time inside and outside the classroom and shifts the decision-making power of learning from the teacher to the student. In this model, teachers will spend little time in class explaining knowledge points in detail. Still, they will spend more time answering students' doubts and broadening their knowledge, requiring students to use their extracurricular time for independent learning. In the flipped classroom model, students' autonomous learning ability has improved [7]. At the same time, through the interaction between teachers and students, students' understanding of knowledge is more thorough, and students' enthusiasm is generally improved [8]. Flipped classroom teaching model is very open, and its internal link can be combined with other methods to achieve a better teaching effect.

Another student-centred teaching method is Team-Based Learning (TBL), in which students are divided into several groups for the sake of learning each other and cooperating to solve problems. Team learning is a kind of active group learning process, by means of the communication and cooperation among group members to complete the learning task and deepen students' impression of knowledge. TBL focuses on the creativity and flexibility of each participant, which can effectively promote students' teamwork spirit, enhance team responsibility, and improve students' autonomous learning ability, communication ability, problem-solving ability and critical thinking ability [9–11]. At the same time, TBL teaching has the advantages of an active classroom atmosphere and interaction between teachers and students, which is unique benefits in improving learning efficiency [9].

With the advent of the information age, network teaching software has emerged in an endless stream, which can be used as a learning platform for students and a platform for teachers to feedback relevant information [12–14]. These platforms can also be used as carriers for the implementation of teaching models. Wechat platform has the characteristics of a short development cycle, easy-operation and personalized customization among all online platforms [15, 16]. With enough flexibility and information capacity, it can be set skillful according to the characteristics of the course, teaching characteristics and objectives. In addition, the platform animation display, games, rankings and other interesting functions enhance students' mastery of professional knowledge, and stimulate learning enthusiasm. At the same time, the backstage can also count the students' learning situation and give real-time feedback to the teacher in order to dynamically adjust the teaching plan.

Studies have shown that flipped classroom, TBL and WeChat platform have all been applied in teaching clinical courses, and have received good evaluation and feedback [17–21]. However, there needs to be more relevant research on the case of the combined application of the three in biochemistry teaching. Therefore, in this study, we explored the teaching effect of the blended teaching model of flipped classroom combined with TBL based on the WeChat platform in a biochemistry course, aiming to study the influence of this new teaching model on the knowledge mastery ability, autonomous learning ability and problem-solving ability of clinical undergraduates.

## Materials and methods

### Experimental design

A mixed research method of quasi-experimental research design and descriptive qualitative research was used to establish a traditional teaching group and a blended teaching group.

### Study participants

A total of 68 freshmen from two classes of nursing and medical imaging technology in Nantong University were selected as the research subjects. The inclusion criteria were:(1) no abnormal physical or mental symptoms at present; (2) full-time medical undergraduates; (3) No experience of using WeChat platform for medical professional course learning before this experiment; (4) before this experiment, there was no statistical difference in learning level and learning ability between two classes; (5) Voluntary participation in this study signed informed consent. The control group selected 32 students and adopted the traditional teaching model; The experimental group selected 36 students and adopted the blended teaching model of flipped classroom combined with TBL based on WeChat platform.

All students are in high school through the national college entrance examination directly into the university, a total of 12 years of education. After the inclusion, the characteristics between the two groups of students (including age, gender and pre-course professional average grade point) were evaluated, and the results showed that the learning ability of the two groups was comparable (*P* > 0.05). They were all taught biochemistry and molecular biology in the second semester of their first year. Both groups of students used the ninth edition of "Biochemistry and Molecular Biology" published by People's Health Publishing House and edited by Zhou Chunyan, and were taught by the same teachers.

### Teaching intervention

#### The control group teaching method

The control group adopts traditional teaching method. Before class, teachers will assign the content to be learned in advance. In class, teachers teach according to the prepared lesson plan and students listen to lectures. After class, teachers assign relevant homework.

#### Flipped classroom based on WeChat platform with TBL blended teaching model

Setting up a teaching team: The biochemistry teaching team is composed of teachers with more than five years of teaching experience. Before teaching, they all received the training of flipped classroom and TBL teaching model and were able to use WeChat platform skillfully.

**Fig. 1:**
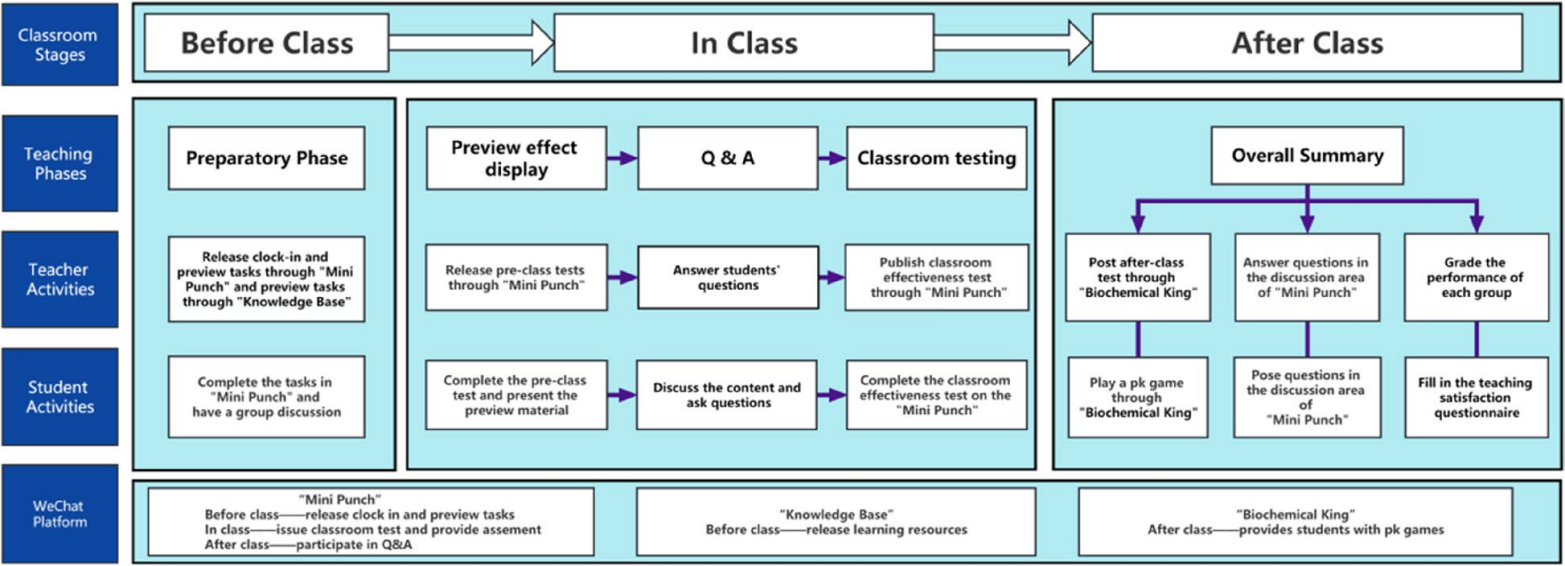
The flow chart of benlded teaching model

Education Reform Implementation: The experimental group of the education reform implementation plan adopts the blended teaching model of flipped classroom combined with TBL based on the WeChat platform, which is carried out before, during and after class respectively (Fig. 1).

- **(1) **The establishment of a hybrid learning team: reasonable grouping is an important prerequisite for team learning. In order to strengthen group learning and achieve the best learning effect, each group has a maximum of 6 students. Therefore, before the class, the teacher will determine the groups according to the average grade point of the students, so that the overall learning ability level of each group is equal. Finally, the students in the experimental group were divided into six groups. Group members were adjusted during the first week in the light of teacher feedback on student performance. In each group, one student is selected as the group leader responsible for group activities. Clear team division of labor ensures the participation of each member and promotes cooperation within the group.
- **(2) **Construction of WeChat Platform of "Biochemical New World": WeChat Platform of "Biochemical New World" includes WeChat mobile phone terminal for students to log in and WeChat management terminal for teachers to manage.
  - **①WeChat mobile phone terminal**
    WeChat mobile phone is the main way for students to access the mobile learning platform. Students can enter the corresponding interface after following the "Biochemical New World" WeChat public account with personal information. WeChat mobile phone terminal mainly includes three sections: "Mini Punch", "Knowledge Base" and "Biochemical King".
  - **②"Mini Punch"**
    "Mini Punch" is a sharing platform and control platform, which promotes the implementation and development of online biochemistry teaching tasks. It can realize the optimization and integration of online and offline resources, deepen the sharing mechanism of data, so as to realize the dynamic monitoring and control of students' biochemistry learning progress. In this process, the actual learning quality and learning effectiveness of students can also be scientifically evaluated with the help of effective backstage data.
  - **③"Knowledge Base"**
    "Knowledge Base" is composed of two parts: teaching content and self-test. The teaching content covers the materials required for teaching the courses, such as Syllabus, lesson plans and weekly calendars for students to carry out preview and review.
    The self-test system is also an indispensable part. In the examination system, teachers regularly publish self-test questions in the WeChat public account according to the teaching progress. Students scan the code for self-examination to achieve the effect of timely review and full digestion of knowledge points.
  - **④"Biochemical King"**
    The "Biochemical King" platform provides students with pk games related to biochemistry. Students can check and fill gaps in knowledge points in pk to stimulate students' challenging psychology and promote student interest in biochemistry courses through competition.
- **(3) Pre-class preparation**
  According to the teaching objectives, teachers determine the contents of difficulties and key points, and then issue clock-in and preview tasks before class in the "Mini Punch" program, in the end, publish the learning resources of this chapter in the "Knowledge Base", including PPT, video resources, massive open online course resources and related cases, etc. Each group leader take charge of two tasks before class. The first task is to organize the team members preview in the "Mini Punch" after following the Wechat public account "Biochemical New World", another is to conduct group discussion on the cases in the "Knowledge Base" and submit the discussion results to the teacher the day before class.
- **(4) Class teaching**
  The classroom design of blended teaching in classroom teaching is mainly divided into the following three parts:
  - **①**In the pre-test session The teacher will issue the pre-class test in the "Mini Punch", which will be completed by the students within 10 min. According to the test result, the teacher organizes teaching activities and selects a group representative to take the stage to display the content of autonomous learning. After the presentation, the teacher will supplement the incorrect and omissive knowledge points.
  - **②**In the Q & A session The teachers were undertaken to address problems that cannot be solved by pre-class and inter-group discussions.
  - **③**In the post-Test class The teacher will issue an after-school test in the "Mini Punch". This test contains more comprehensive topics and is completed by the students in class, in order to test the teaching effect of this class and the students' mastery of knowledge in this chapter.
    Teachers organize teaching activities, and student representatives of each group explain the content of autonomous learning in turn. After the end, teachers and students ask questions or discuss, and students of each group complete the demonstration of autonomous learning results, by the teacher to conduct a brief evaluation, content summary.
- **(5) Overall summary**
  In order to help students fully consolidate what they have learned in class, teachers release the content of the phased pk answer game "Biochemical King" through WeChat after class. Students can pay attention to the WeChat public account to enter the game, consolidate the knowledge points of this chapter by launching two PK answers with other students. In view of the questions that cannot and do not understand in the process of answering questions, students ask questions to the teacher through the discussion area where they can punch in. Finally, the teacher makes a brief evaluation of each group according to the preview before the group class and the classroom discussion. The group students use Mini Punch combined with "questionnaire star" to give questionnaire feedback on the classroom teaching situation, to promote the smooth development of teaching reform.
  After class, students complete the task of sorting out the key notes in class. Pay attention to the WeChat public account "Biochemical New World" to complete the task of "Mini Punch", select the teaching content of "Knowledge Base" to review and self-test at the same time, scan the code "Biochemical King" and classmates pk.

### Measurement of students’mastery of knowledge

The two groups of students will take the written examination at the end of the semester. The questions of the written examination are divided into three levels of difficulty: easy, medium and hard. The 100 questions have a total of 100 points (1 point/question), among them, there are 60 simple topics, 30 medium topics and 10 difficult topics.

### The overall satisfaction survey of teaching

The overall teaching satisfaction questionnaire consists of three parts: course arrangement, teaching quality and teaching style, with a total of 21 questions: the evaluation grade is divided into satisfactory, general and unsatisfactory; the overall satisfaction rate of teaching = the number of cases with satisfactory evaluation/the total number of cases × 100%. The questionnaire in this study was compiled with Questionnaire Stars.

### Qualitative evaluation-semi-structured interview

At the end of the course, a semi-structured interview was conducted to investigate the evaluation from the mixed teaching group on the application of the mixed teaching model about biochemistry and molecular biology. Considering the gender, age, and scores of the students, purposive sampling was carried out in the blended teaching group to ensure the diversity of opinions. The interview would be over until no new topic or information come up.

In the sake of fully understanding the teaching relating to the blended teaching model of the flipped classroom on WeChat platform combined with TBL and the real experience from students, the research team implemented a preliminary interview with two students, and then the final interview outline was determined:(1) How do you feel about the blended teaching model of biochemistry and molecular biology? (2) Do you think your learning condition has changed compared with before? (3) What are the favorable suggestions for the application of hybrid teaching model in biochemistry and molecular biology?

A researcher well versed in interviewing techniques was assigned to perform the interview independently. Interviews are conducted in a quiet and relaxing session during the week following the course in order to avoid errors as much as possible. Each interview lasted about 20 min. The students' conversations were recorded, and the research team promised to keep the conversations confidential. A recording of the interview was transcribed verbatim into words within 24 h of the conversation.

### Data analysis

RStudio software (version 4.3.1) was used for data input and data analysis with the aid of the R packages "stats", "car", "doBy", "ggplot2". Independent samples t-tests were applied for quantitative data, and chi-square tests were served to compare qualitative data. *P* < 0.05 on behalf of the difference was statistically significant.

## Results

### Characteristics of participants

The blended teaching group consists of 36 students aged 20–25 (21.92 ± 0.907)years old in class 221 Nursing. The traditional teaching group has 32 students in class 221 imaging, aged from 21 to 24 (21.94 ± 0.859)years old. Before the beginning of the course, we surveyed their academic scores in the previous semester. The average grade point of the Blended teaching group was (2.793 ± 1.031), while that of the Traditional teaching group was (2.952 ± 0.956). There was no significant difference in demographic characteristics between the two groups (*p* > 0.05)(Table 1).

**Table 1:** Comparison of demographic characteristics between the Blended teaching group and Traditional teaching group

Characteristics	Blended teaching group (*n* = 36)	Traditional teaching group (*n* = 32)	Statistics	*P*-value
Age	21.92 ± 0.907	21.94 ± 0.859	*t* = 0.086	0.932^b^
Gender				
Male	17	19	*χ*^*2*^ = *0.236*	0.627^a^
Female	17	15		
Grade Point Average before the course	2.793 ± 1.031	2.952 ± 0.956	*t* = 1.144	0.255^b^

^a^Pearson’s chi-squared test,

^b^Independent-samples *t*-test

### Final exam results for the blended teaching group and traditional teaching group

**Fig. 2:**
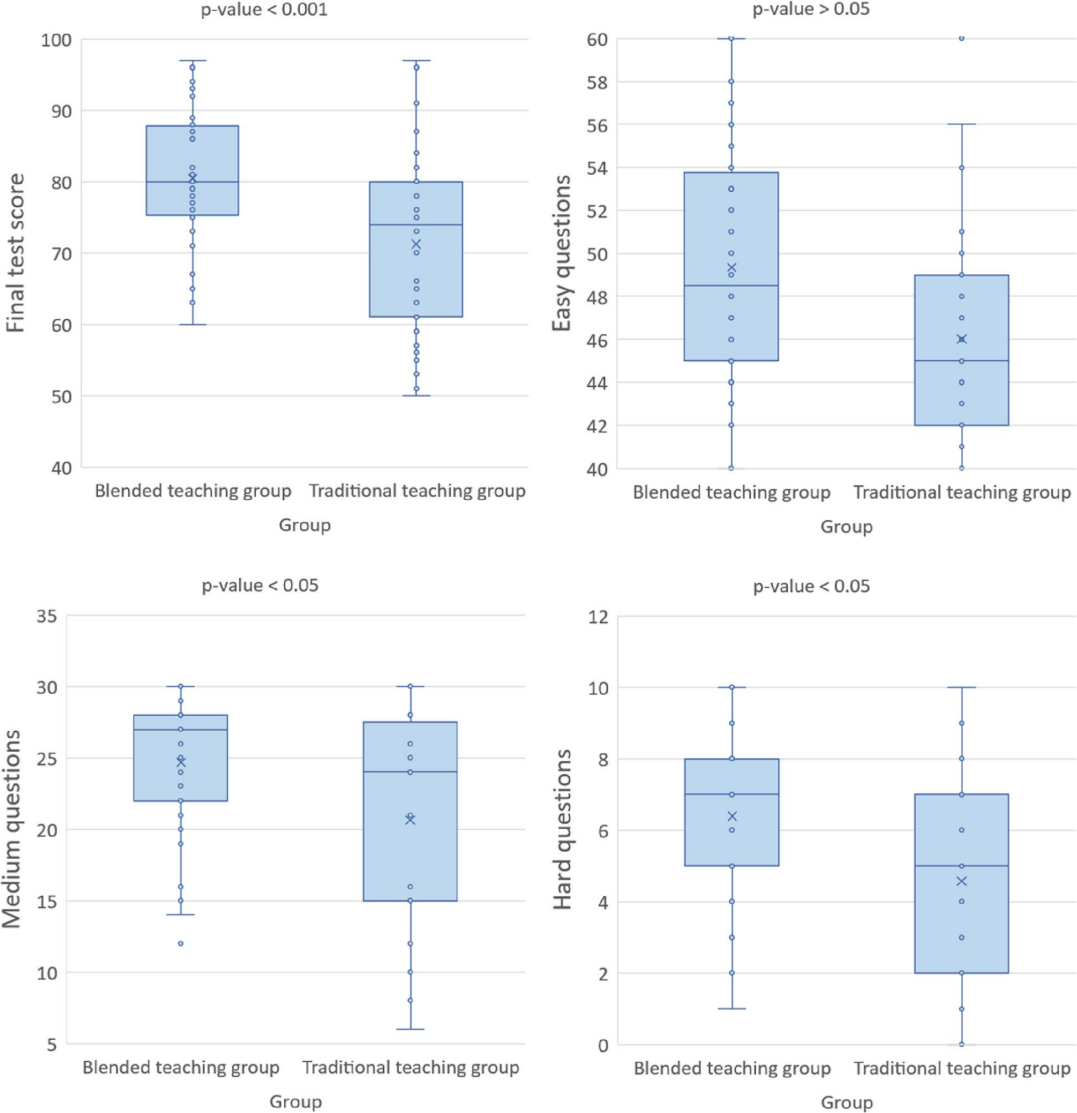
Final exam results for Blended teaching group and Traditional teaching group

In the final exam, the test score showed that students in the blended teaching group scored an average of 84.44 points, while students in the traditional teaching group earned a mean score of 71.22 points. The average score of traditional teaching group was significantly lower than  the blended teaching group’s in the final exam (*P* < 0.001). Among them, there was no significant difference in the score of easy questions between the two groups (*P* > 0.05). In the medium difficulty questions, the average score of the mixed teaching group was 24.69, significantly higher than that of the traditional teaching group (20.66, *P* < 0.05). In terms of difficult questions, average score in the blended teaching group were still higher than those in the traditional teaching group (Fig. 2).

### Satisfaction survey

After the questionnaire survey, 68 students completed the questionnaire survey, and 68 valid questionnaires were recovered, with a total completion rate of 100%. As shown in Table 2, it can be seen that the overall satisfaction of the blended teaching group on course arrangement, teaching quality and teachers' teaching style is higher than that of the traditional teaching group, and the difference between the two groups is statistically significant (*P* < 0.05).

**Table 2:** Comparison of Blended teaching group and Traditional teaching group on the overall satisfaction of teaching

Group	N	Course Arrangement	Teaching Quality	Teaching Style
Blended teaching group	**36**	31(86.11%)*	34(94.44%)*	35(97.22%)*
Traditional teaching group	**32**	**24(75.00%)**	**26(81.25%)**	**29(90.63%)**

^*^The difference between the observation group and the control group was statistically significant, *P* < 0.05

### Qualitative data analysis

By summarizing the interview results, we can divide into the following three aspects: (1) stimulating learning interest (2) improving autonomous learning ability (3) suggestions for perfecting teaching model.

#### Theme 1: Stimulate interest in learning

Compared with traditional teaching, blended teaching successfully combines online with offline teaching resources (offline classroom teaching, online "Biochemical King" game, etc.) to attract students' attention. Especially, the animations in the WeChat public platform transform boring knowledge into vivid, exciting and easy-to-understand stories, which can deepen their understanding of knowledge and further stimulate their interest in learning.

"The previous teaching methods also emphasized the importance of preview, but due to the lack of a certain guidance, our preview efficiency was low. In blended teaching, teachers provide corresponding teaching videos and cases, particularly in animations before class. After them, students can accept new knowledge more quickly with the problems encountered in preview." (S1).

"In our group, everyone will have a different division of work, despite each class will be around a problem for discussion. After class, for our common object, communicating with each other in the same team and exploring together unknown things really makes us feel a sense of achievement." (S2).

"The Biochemical King game is very attractive to me. When we compete in the game, everyone also care about the performance ranking. We interact within the group and help one another, which contributes our members to upgrade in the class." (S3).

#### Theme 2: Improving self-directed learning ability

Teachers assign relevant preview tasks before class so that students can clarify the learning objectives of the course and be assessed their autonomous learning ability through the pre-class test. After class, students can summarize the learning content and existed deficiencies by going in for the "Biochemical King" academic competition. In addition, "Biochemical King" built-in extensive data analysis, according to their own competition situation, students can adjust their learning strategies to enhance the memory of basic knowledge and the understanding of difficult knowledge.

"The case analysis and preview tasks before class will urge me to take the initiative to preview and find out practical knowledge points. The tests before and after class make learning more purposeful and well planned, stimulating my enthusiasm for learning.”(S4).

"This teaching method makes me more involved in team study and class participation. I become more motivated to learn, independently find solutions to questions and discuss them with other team members. During I was studying in a team, my self-esteem and sense of accomplishment make me study harder and take the initiative to master knowledge." (S5).

"After the teacher released the "Mini Punch" and preview tasks before class, I consciously fulfilled tasks in case to fall behind the teacher's progress in class as well as affect the learning progress of other students in the group." (S6).

#### Theme 3: suggestions for perfecting teaching model

Aiming at the blended teaching model in biochemistry, students put forward some suggestions positively.

" Previewing before class is very helpful for improving stable academic performance, but sometimes it takes a lot of time and brings some burdens. I wish teachers could upload more concise materials and exercises. " (S7).

"Under this teaching model, I feel that I have a higher mastery of this course than other subjects’, and I have a greater interest and enthusiasm in this course. However, mentioning the preview presentation before class, students are often at a loss. We were inexperienced in presentation and needed more tutorial guidance." (S8).

## Discussion

In China, the teaching concept of higher education has gradually shifted from teacher-centered to student-centered, and the teaching methods must be adjusted accordingly [22–24].Traditional teaching methods can no longer meet the needs of modern medical education. As a required course for medical students, biochemistry covers more tedious knowledge points, which directly leads to the learning inefficiency of students [25, 26]. Many courses now take the form of flipped classroom due to the COVID-19 pandemic, where students get learning information online before class and teachers have better strategies for enhancing remote student engagement through active learning. At the same time, studies have shown that the TBL method can encourage the further development of an individual's communication skills and problem-solving ability during team as a whole. The previous period of correlation analysis and evaluation employed in covid-19 has brought machine learning, decision trees and ANN algorithms into the horizon of researchers, we have reasons to believe that these algorithms and methods can also be applied in online teaching evaluation [27–32].

In our research, the flipped classroom teaching model combined with TBL is implemented based on our self-developed WeChat platform. A blended teaching model based on WeChat platform is an important model and development direction of educational practice reform. Due to the advantages of low development cost, less consumption flow, and small memory space, WeChat public platform has been adopted by more and more educators to establish a personalized teaching platform [33, 34]. By integrating the teaching platform with WeChat small program, the teaching model will undergo significant quantum leap and adapt to the requirement of modern education.

WeChat public platform promotes the development of teaching model and the transformation of closed classroom into open classroom. In the limited teaching time, the process of knowledge internalization is strengthened through the group discussion of students, then the learning quality is enhanced. The teaching resources in the menu bar of "Knowledge Base" expand the coverage of teaching content, and students arrange their convenient time for learning. Animation take the abstract content of biochemistry into shape, helping students to memory and comprehend profound knowledge. Micro-lessons make full use of fragmented time to greatly improve students learning efficiency and learning flexibility. The WeChat mini program "Biochemical King" offers after-school quizzes in which students vie with each other, which has dramatically boosted students' motivation. Students can also sort out the wrong questions and constantly improve the knowledge system to enhance the efficiency of learning. With the application of big data and ANN algorithms, teachers can monitor the students' understanding degree of certain knowledge in the background management system so as to regulate the key and difficult points in the teaching process in real time. Unlike the previous teaching of all knowledge points, the blended teaching method can significantly improve the teaching efficiency of teachers as well as the learning efficiency of students.

The results of this study show that the final examination mean scores of students in the blended teaching method are generally higher than those of students in the traditional teaching group, which further demonstrates that the blended teaching method based on WeChat platform can better raise the scores of students, especially in the medium difficulty questions and hard difficulty questions (Table 2). However, the two groups had no significant difference in the simple difficulty questions (*P* > 0.05). The possible reason is that the simple difficulty questions are relatively easier to remember and the answer accuracy is higher. In the interview, students also reflected that the teaching model of TBL combined with flipped classroom can guide them to find learning problems and resort to team, which has a very positive significance for them to enter the clinical stage in the future.

According to the questionnaire of students' satisfaction with blended teaching, students were delighted with the curriculum arrangement, teaching quality and teachers' teaching style, as well as the organization form and assessment method of this teaching form, and considered that this teaching model was excellent. The blended teaching model based on WeChat platform has greater feasibility and suitability in biochemistry teaching. Students showed that learning on WeChat platform not only assists them to understand and master the content of the course systematically but also stimulates their interest in autonomous learning, upgrades their ability to discover and solve problems, and enhances their comprehensive ability, such as team cooperation. The vast majority of students hold a positive attitude towards the blended teaching model. In addition, some students also hold their own views on the blended teaching model. The main problem is that this teaching model takes up a lot of extracurricular time, and students are not accustomed to telling knowledge by themselves, which also provides valuable suggestions for our future improvement of teaching model and perfection of after-class materials.

To sum up, the teaching model of flipped classroom combined with TBL based on WeChat platform in our study is centered on student groups. The self-developed WeChat public platform of biochemistry provides students with pre-class materials, exciting animations, and "Biochemical King" games to strengthen students' understanding and application of biochemical knowledge and solves common problems encountered by students in the learning process through online interaction. Student groups help each other offline, make common progress, break through key points and difficulties, and further level up students' autonomous learning ability, teamwork ability and problem-solving ability, which are essential skills and qualities in future clinic work. Based on the interviews and in-depth analysis of biochemistry teaching scores, we found that compared with the traditional teaching method, students would increase their interest in biochemistry learning and enhance their overall ability exercise through the blended teaching method, which indicates that the teaching model of flipped classroom combined with TBL based on WeChat platform is very appropriate for biochemistry teaching. In general, the teaching model of flipped classroom combined with TBL based on WeChat platform is an effective way to promote learning, and online and offline complement each other, which is worthy of promotion and trial in the teaching of other disciplines in the future.

## Data Availability

Due to concealment involving participants, privately anonymous datasets will be sent to by reasonable request corresponding author.
